# Infarct volume after glioblastoma surgery as an independent prognostic factor

**DOI:** 10.18632/oncotarget.11482

**Published:** 2016-08-22

**Authors:** Stefanie Bette, Benedikt Wiestler, Johannes Kaesmacher, Thomas Huber, Julia Gerhardt, Melanie Barz, Claire Delbridge, Yu-Mi Ryang, Florian Ringel, Claus Zimmer, Bernhard Meyer, Tobias Boeckh-Behrens, Jan S. Kirschke, Jens Gempt

**Affiliations:** ^1^ Department of Neuroradiology, Klinikum rechts der Isar, Technische Universität München, Munich, Germany; ^2^ Department of Neurosurgery, Klinikum rechts der Isar, Technische Universität München, Munich, Germany; ^3^ Department of Neuropathology, Klinikum rechts der Isar, Technische Universität München, Munich, Germany

**Keywords:** glioblastoma, infarct volume, karnofsky performance score, overall survival

## Abstract

Postoperative ischemia is associated with reduced functional independence measured by karnofsky performance score (KPS), which correlates well with overall survival. Other studies suggest that postoperative hypoxia might initiate infiltrative tumor growth. Therefore, aim of this study was to analyze the impact of infarct volume on overall survival and progression free survival (PFS) of glioblastoma patients.

251 patients with surgery for a newly diagnosed glioblastoma (WHO IV) were retrospectively assessed. Pre- and postoperative KPS, date of death/last follow-up and histopathological markers were recorded. Pre- and postoperative tumor volume and the volume of postoperative infarction were manually segmented.

A significant correlation of infarct volume with postoperative KPS decrease (*P* = 0.001) was observed. Infarct volume showed a significant impact on overall survival (*P* = 0.014), but not on PFS (*P* = 0.112) in univariate analysis. This effect increased in the subgroup of patients with near-total tumor resection (> 90%) (overall survival: *P* = 0.006, PFS: *P* = 0.066). Infarct volume remained as an independent prognostic factor for overall survival in multivariate analysis (HR 1.013 [1.000–1.026], *P* = 0.042) including other prognostic factors (age, extent of resection, postoperative KPS).

Postoperative infarct volume significantly correlates as an independent factor with overall survival after glioblastoma surgery. Besides the influence of perioperative infarction on postoperative KPS, postoperative hypoxia might also have an effect on tumor biology initiating infiltrative growth and therefore impaired survival.

## INTRODUCTION

Glioblastoma (GB) is the most frequent malignant brain tumor in adults and, despite recent advances in therapy, still has a poor overall survival with a 5-year survival rate of only 5% [1, 2]. Extent of resection and functional independence after surgery are well-known prognostic factors and play an important role for overall survival [3–6]. Therefore, maximum tumor resection and preservation of motor and language function are aims of surgery [5, 6]. About 5–19% of patients show permanent or transient neurological deficits after glioma surgery [7–9]. These postoperative neurological deficits usually can be explained by the following: surgical tissue damage, hemorrhage, venous congestion, vessel occlusion, and consecutive infarction [10]. A recent study showed that postoperative ischemic changes occur in 31% of newly diagnosed and 80% of recurrent glioma and have a high impact on neurological outcomes [10]. Due to postoperative changes, formation of scarred tissue, altered vascular supply, and post radiogenic changes, the risk of infarction is increased in patients with recurrent surgery [7, 11]. Karnofsky performance score (KPS) is an important prognostic factor for patients with glioblastoma and is routinely used to assess the functional independence of tumor patients perioperatively and on follow-up examinations [12–14]. Postoperative ischemic changes are associated with neurological deficits and consecutive reduction of functional independence, as has been previously assessed by qualitative analysis [10, 15]. Studies showed that impaired postoperative KPS and new postoperative neurological deficits caused by ischemic changes are associated with shorter overall survival [16, 17]. Another recent study showed that postoperative ischemia was associated with a diffuse tumor recurrence pattern, which however showed no correlation to overall survival in this patient cohort but suggests hypoxia as a factor for tumor growth [18].

Therefore, the aim of this study was to analyze the impact of postoperative ischemic changes and postoperative infarct volume on KPS and overall survival/progression free survival after glioblastoma surgery.

## RESULTS

### Patient population

251 consecutive patients (100 female, mean age at initial diagnosis 63.1 years) with surgery for a newly diagnosed glioblastoma (WHO IV) were retrospectively included in this study (Table 1). 10 patients received previous stereotactic biopsy, 1 of these 10 patients also received radiotherapy prior to surgery at the time of data acquisition. The median preoperative KPS was 80 (IR 70–90), and the median postoperative KPS was 70 (IR 60– 90). During follow up, 178 out of the 251 patients died. Median overall survival was 13.0 months (95% CI 11.4–14.6) after initial diagnosis of a glioblastoma. 221/251 patients presented with recurrent disease. Recurrent disease was proven by histopathological analysis in 70/221 cases, according to the RANO criteria in 49/221 cases, by advanced imaging methods including O-(2-[18F]-Fluoroethyl)-L-Tyrosine-Positron Emission Tomography in 22/221 cases and by death in 80/221 cases. Median PFS was 5.8 months (95% CI 4.9–6.7). Histopathological analysis revealed MGMT-methylation in 67/167 cases and IDH1/2-mutation in 3/133 cases.

**Table 1:** Baseline patient and tumor characteristics

Age at date of initial diagnosis	63.1 (+/−13.2)
Sex, female	100/251 (39.8%)
KPS preoperative	80 (70–90)
KPS postoperative	70 (60–90)
Postoperative KPS decrease	99/251 (39.4%)
MGMT-methylation	67/167 (40.1%)
Death during FU	178/251 (70.9%)
OS after ID	13.0 months (95% CI 11.4–14.6)
Recurrent disease	221/251 (88.0%)
PFS after ID	5.8 months (95% CI 4.9–6.7)

normally distributed variables shown as mean +/− standard deviation, non-normally distributed as median (interquartile range); f, female; FU, follow up; OS, overall survival, ID: initial diagnosis.

### Postoperative ischemia and volumetric measurements

Postoperative ischemic changes, including also minor areas with restricted diffusion surrounding the resection cavity, were present in 226/251 postoperative MRI scans. Small rim-like infarctions surrounding the resection cavity were present in 78/251 patients. Median infarct volume was 2.0 cm^3^ (IR 0.6–6.9) (Table 2).

**Table 2:** Postoperative ischemia and measurements of pre- and postoperative tumor volume and volume of postoperative infarction

Postoperative ischemia	226/251 (%)
Postoperative infarct volume	2.0 cm^3^ (0.6–6.9)
Preoperative tumor volume	30.7 cm^3^ (12.9–54.5)
Postoperative tumor volume	0.0 cm^3^ (0.0–0.9)
Complete tumor resection	127/251 (50.6%)
Tumor resection > 90%	95/251 (37.8%)
Tumor resection < 90%	29/251 (11.6%)

Non-normally distributed variables shown as median (interquartile range).

Median preoperative tumor volume was 30.7 cm^3^ (IR 12.9–54.5), while the median volume of residual tumor after surgery was 0.0 cm^3^ (IR 0.0–0.9). Complete tumor resection was achieved in 127/251 cases (50.6%), while 95/251 (37.8%) had near-total tumor resection (< 10% of initial tumor volume) and 29/251 (11.6%) subtotal tumor resection (> 10% of initial tumor volume). Postoperative infarct volume significantly differed between patients with total / near total (> 90%) tumor resection (median 2.5 cm^3^ [IR 0.6–7.2]) and subtotal (< 90%) tumor resection (median 1.0 cm^3^ [IR 0.0–1.8]) (*P* = 0.004).

No significant correlation of infarct volume to MGMT-methylation status was observed. Median infarct volume was 1.6 cm^3^ (IR 0.4–7.6) in cases without MGMT-methylation and 2.3 cm^3^ (IR 0.6–6.4) in cases with MGMT-methylation (*P* = 0.572).

### Postoperative karnofsky performance score (KPS)

**Figure 1:**
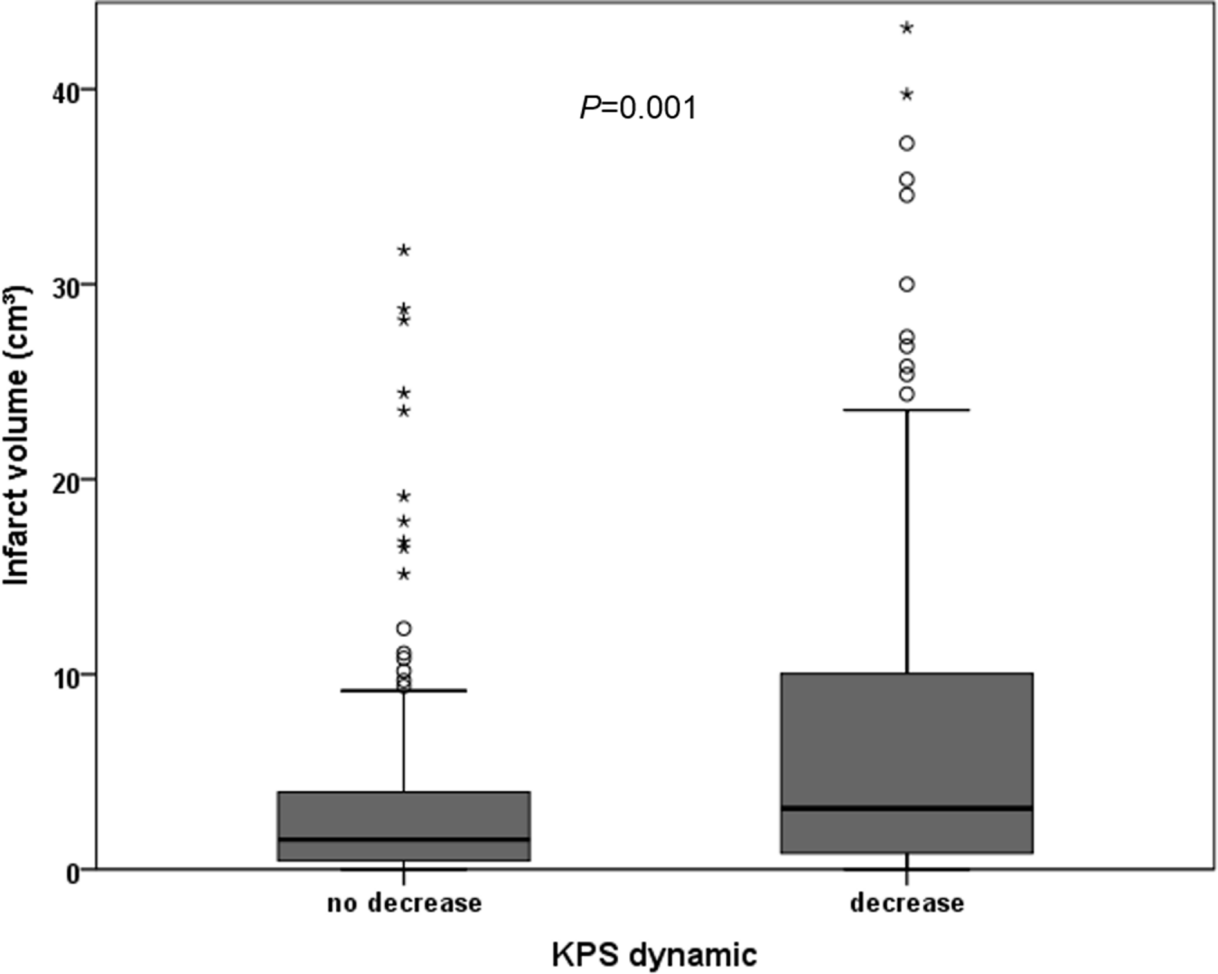
Distribution of infarct volume in correlation to postoperative KPS dynamic

Infarct volume was significantly higher in patients with decreasing KPS after surgery (*P* = 0.001). Median infarct volume was 3.1 cm^3^ (IR 0.8–10.1) in patients with decreasing KPS and 1.5 cm^3^ (IR 0.4–4.0) in patients with stable or increasing KPS after surgery. A significant correlation between postoperative KPS decrease and infarct volume (*r* = 0.213, *P* = 0.001) was observed. Figure 1 shows the distribution of infarct volume in patients with or without postoperative KPS decrease.

### Overall survival

#### Univariate model

**Figure 2:**
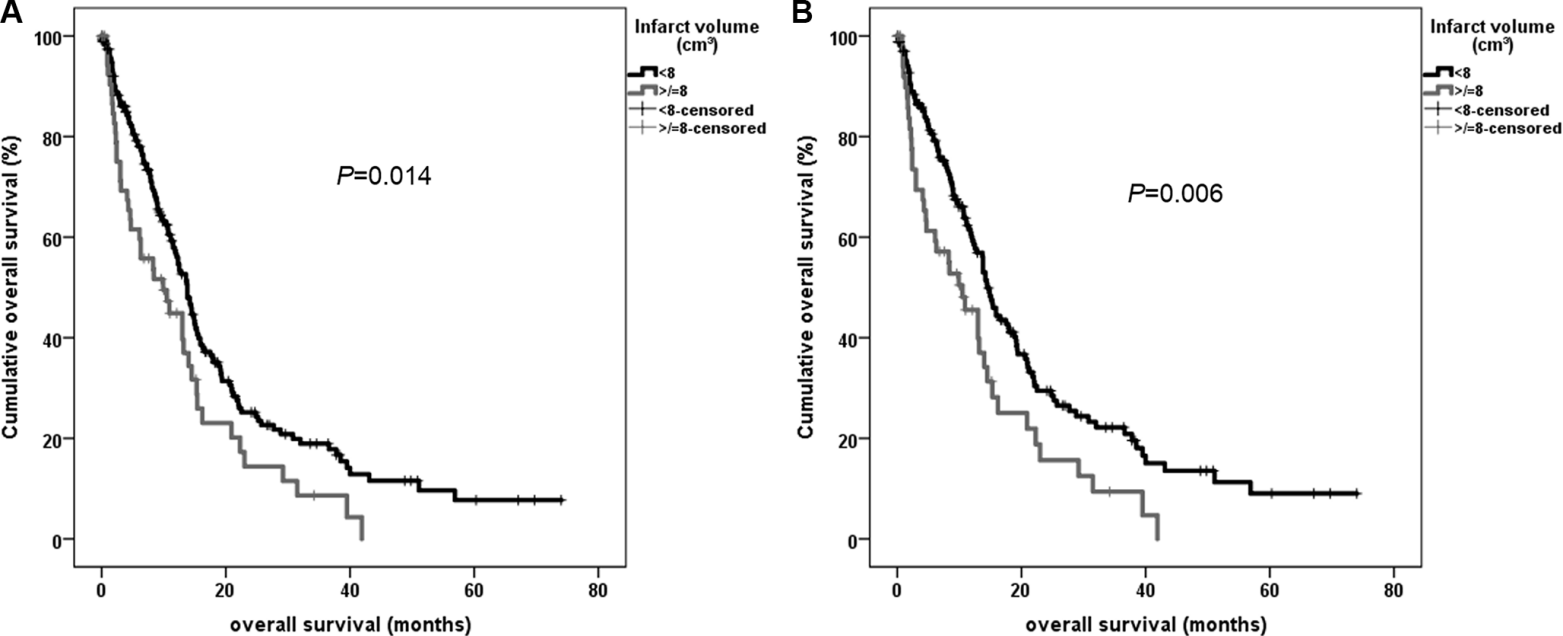
Univariate overall survival analysis using Kaplan-Meier for dichotomized infarct volume in all patients (A) and for patients with total and near-total tumor resection (> 90%) (B)

Univariate analysis using Kaplan-Meier estimates revealed that the following parameters show a significant impact on overall survival: extent of resection (*P* < 0.001) and postoperative KPS decrease (*P* = 0.001). Significantly impaired overall survival was observed for patients with an infarct volume >/= 8 cm^3^ (*P* = 0.014) (Figure 2).

Analyzing only patients with total and near-total tumor resection dichotomized infarct volume (*P* = 0.006) showed also a significant correlation to overall survival (Figure 2). This effect remained significant in the subgroup analysis excluding patients with small rim-like ischemic changes (*P* = 0.022) and also in the groups of patients with total and near-total tumor resection (*P* = 0.008) (Supplementary Figure S1).

MGMT-methylation status did not show significant impact on overall survival in this cohort, however the MGMT-methylation status is missing in many cases (*P* = 0.369).

#### Multivariate model

Multivariate survival analysis was performed using a proportional hazards regression analysis (Cox regression model) for the following parameters: age at initial diagnosis, postoperative KPS (> 80, </= 80), extent of resection (complete, >/= 90% vs. < 90%), infarct volume (cm^3^) for all patients and in a second step only for patients with tumor resection > 90% (extent of resection as variable was excluded in this analysis).

The multivariate model revealed the known important variables like age at initial diagnosis (*P* < 0.001, Hazard ratio (HR): 1.044 [95% CI 1.030–1.059]) and postoperative KPS (*P* < 0.001, HR: 0.479 [0.331–0.693]) as significant variables. The hazard ratio for tumor resection < 90% instead of complete resection was 2.436 [1.538–3.857] (*P* < 0.001). The hazard ratio for infarct volume was 1.013 [1.000–1.026] (*P* = 0.042).

Including only patients with tumor resection > 90% hazard ratio as well as significance level of infarct volume increased in multivariate analysis (HR 1.019 [1.005– 1.032], *P* = 0.005), beneath other known significant parameters like age (HR 1.051 [1.035– 1.068], *P* < 0.001) and postoperative KPS (HR 0.481 [0.327– 0.707], *P* < 0.001).

Excluding patients with rim-like infarctions infarct volume just missed statistical significance in multivariate analysis for all patients (*P* = 0.063, HR: 1.013 [0.999– 1.027]; age: *P* < 0.001, HR: 1.047 [1.030–1.065], postoperative KPS: *P* = 0.016, HR: 0.562 [0.352–0.900], extent of resection: *P* = 0.001, HR: 2.533 [1.433–4.477]), but showed significant results in the subgroup analysis of patients with tumor resection > 90% (*P* = 0.007, HR: 1.019 [1.005–1.033]) beneath other significant parameters such as age (*P* < 0.001, HR: 1.056 [1.036–1.076]) and postoperative KPS (*P* = 0.022, HR: 0.572 [0.355–0.922].

### Progression free survival

#### Univariate model

**Figure 3:**
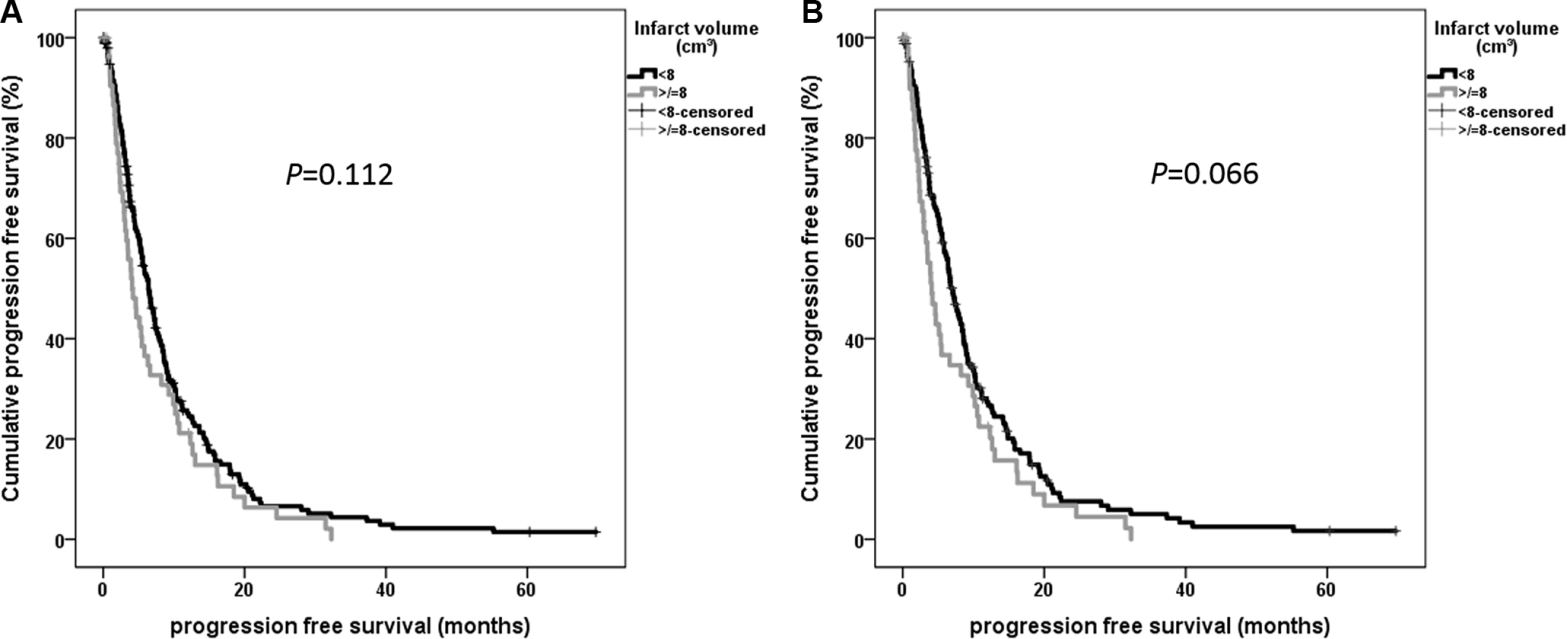
Univariate progression free survival analysis using Kaplan-Meier for dichotomized infarct volume for patients with total and near-total tumor resection (> 90%)

Extent of resection showed significant impact on PFS in univariate analysis (*P* < 0.001). Postoperative KPS decrease (*P* = 0.068) as well as dichotomized infarct volume for all patients (*P* = 0.112) and for patients with near-total tumor resection (*P* = 0.066) missed statistical significance (Figure 3).

#### Multivariate model

Multivariate survival analysis was performed analogous to overall survival including the following parameters: age at initial diagnosis, extent of resection, postoperative KPS (> 80, </= 80) and infarct volume (cm^3^). Age (HR 1.025 [1.014–1.037], *P* < 0.001), postoperative KPS (HR 0.686 [0.498–0.944], *P* = 0.021) and extent of resection (< 90% vs. complete resection; HR 2.349 [1.504–3.667], *P* < 0.001) showed significant impact on PFS, infarct volume was not shown as prognostic factor for PFS (HR 1.009 [0.997–1.021], *P* = 0.158). Similar results were observed for the subgroup of patients with near-total tumor resection (infarct volume: HR 1.010 [0.997–1.023], *P* = 0.131).

## DISCUSSION

Postoperative infarct volume after glioblastoma surgery is an independent prognostic factor for postoperative functional independence and for overall survival, but not for progression free survival. These data suggest that reduction of infarct volume during gross total tumor resection in glioblastoma surgery is desirable, not only to avoid neurological deficits but to improve overall survival as well. Further these data show that postoperative ischemia causing hypoxia in the surrounding tissue might be important for tumor growth.

The incidence of postoperative ischemic changes varies between 19–80% depending on type of tumor, tumor grading, or previous surgeries and has a high impact on postoperative neurological status [10, 19–22]. To prevent ischemic postoperative changes associated with neurological deficits, it is important to understand its cause. Previous studies classified postoperative ischemic changes in an attempt to refer the cause of ischemia [10, 19]. Arterial infarctions corresponding to diffusion restriction in areas of direct branches of the anterior/middle/posterior cerebral artery are probably caused by direct or indirect vessel damage or occlusion. Possible surgical mechanisms are pressure by the spatula or direct damage by bipolar coagulation [10, 23]. Infarction in terminal branches is possibly caused by direct damage of smaller vessels [10]. Venous infarctions including hemorrhage were assumed to be direct or indirect damage of veins causing a congestive ischemic lesion [10].

In this study the volume of postoperative infarction was measured. In a way that differs from previous studies, small areas with restricted diffusion surrounding the resection cavity were measured and also classified as postoperative infarction. This explains the high incidence of ischemic changes in this study of about 90%.

A recent study showed that the incidence of postoperative ischemia was 80% in recurrent glioma compared to 31% in newly diagnosed glioma [10]. This is explained by the presence of scar tissue, by different postoperative vascularization patterns, by vessel obliteration and reorganization by radiotherapy, which is also used in therapy for arteriovenous malformation [10, 24]. In this study we only included patients with initial tumor diagnosis and excluded patients with previous surgery to avoid bias to overall survival analysis. Further studies with investigation of the distribution of infarct volume between patients with or without recurrent surgery might have to be performed.

Reduced functional independence classified by the KPS is a known prognostic factor in patients with glioblastoma [13, 14]. Many factors contribute to the KPS such as age, comorbidities, and neurological deficits, whether caused by the tumor or by surgical intervention [7, 14, 25]. Postoperative decrease in KPS can occur due to many factors such as edema, hemorrhage, or structural damage. Ischemic lesions were shown to have a high impact on deterioration of postoperative neurological status and KPS [10]. Studies also showed that ischemic changes are associated with postoperative neurological deficits and reduced KPS [10].

In accordance with these mentioned studies also in this study, worsening of postoperative performance status correlates significantly to ischemic changes. However this study also showed that not only the presence of ischemic changes, but also the volume of ischemic changes correlates to postoperative KPS, suggesting that reduction of postoperative infarct volume is important for postoperative functional independence of glioblastoma patients.

The influence of many factors on survival of glioblastoma patients has been well studied in the last years. Besides molecular markers that are important for responsiveness to chemotherapy and for overall and progression free survival [26, 27], one important prognostic factor is patients' age. The less favorable outcome in elderly patients is associated with different tumor biology, comorbidities, and different therapy strategies [25, 28, 29]. Other important prognostic factors are extent of resection and pre- and postoperative KPS [5, 6, 13, 14, 16, 17].

The results of this study are in common with these previous results and reveal age, extent of resection, and postoperative KPS as the strongest prognostic factors.

Besides these important factors, the postoperative infarct volume also shows an impact on overall survival in glioblastoma patients in multivariate analysis independently of worsening of postoperative KPS. Hypoxia is known to be a mediator of invasive tumor growth also in glioblastoma [30–33]. It was recently shown that ischemia after glioblastoma surgery correlated to an infiltrative and diffuse tumor recurrence pattern which is in common with the results of our study [18]. However in this previous study no impact on overall survival of postoperative infarction was shown, which might be explained by the lower patient number [18]. The results of this study suggest in common with this previous study that postoperative hypoxia could have an influence on tumor biology, tumor growth and overall survival. However of note, the prognostic value of postoperative infarct volume is low compared to the impact of extent of resection (HR 2.436 compared to 1.013 of infarct volume). PFS also differed between patients with or without large infarct volumes, but missed statistical significance in this study cohort. This might be explained by the fact that PFS is in contrast to overall survival susceptible to bias depending on adjuvant therapy, surveillance intervals and retrospective data analysis. Significance of postoperative infarct volume on overall survival increased in patients with total or near-total tumor resection only. This can be explained by the lower infarct volumes in subtotal tumor resections due to less (possible) aggressive surgery and the poor survival of patients with residual tumor. These results suggest that the reduction of infarct volume is an important prognostic factor especially for patients with near-total or total tumor resection.

Postoperative hemorrhage might also have a high impact on outcome and survival, which might introduce a bias in these results as the volume of hemorrhage was not measured in this study. The impact of postoperative bleeding on overall survival in glioblastoma has to be assessed in further studies. Furthermore, it is known that besides the infarct volume, the infarct location has a high impact on neurological outcome [34, 35]. In particular, in eloquent brain areas, small infarctions can cause relevant neurological deficits.

There are several limitations of this study. The main limitation is the retrospective design. Another limitation might be the semiautomatic measurement of tumor and infarct volume. However, manual segmentation and the Cavalieri method have been shown to be precise and reliable [36, 37]. To avoid observer bias, measurement of infarct volume was performed blinded to neurological outcome and overall survival.

In this study, also small ischemic changes surrounding the resection cavity were assessed as ischemia, which might also lead to a bias due to many patients with only very small infarct volumes. However to avoid this bias subgroup analysis excluding patients with these rim-like infarctions was performed and showed similar results.

Another limitation is the fact that methaemoglobin was not registered in manual segmentation of infarct volume. However, here we refer to the method of previous published studies [10, 38].

## MATERIALS AND METHODS

This retrospective, non-interventional single-center study was approved by the local ethics committee (5625–12). The study was in accordance with the ethical standards of the 1964 Declaration of Helsinki and its later amendments [39].

### Patient population

From our database 529 consecutive glioblastoma patients were identified. 251 patients with surgeries for a newly diagnosed glioblastoma (WHO IV) between May 2008 and September 2015 with preoperative and early postoperative MRI, including diffusion-weighted images, were retrospectively included; the others were excluded due to missing diffusion-weighted images or previous surgeries. Clinical data, including pre- and postoperative KPS, the date of initial tumor diagnosis, previous biopsies/irradiation, were documented for each patient. The date of death and the date of last contact in alive patients were recorded. Overall survival and progression free survival (PFS) were calculated from the date of initial tumor diagnosis. Date of recurrent disease was assessed in MR imaging according to the RANO criteria in an interdisciplinary consensus (neurooncology, neurosurgery, neuroradiology, radiotherapy). In cases without ensured recurrence in MRI death was recorded as end point for PFS.

Histopathological analysis was performed according to the WHO criteria of 2007. All patients included in this study were diagnosed with a glioblastoma (WHO IV).

Methylation status of O^6^-methylguanine DNA methyltransferase (MGMT) was assessed in 167/251 cases, Isocitratdehydrogenase 1/2- (IDH1/2-) mutation in 133/251 cases.

### Magnetic resonance imaging

MRI scans were performed in the Department of Neuroradiology at a 3 Tesla MRI scanner, either Philips Achieva or Philips Ingenia (Philips Medical Systems, The Netherlands B.V.) or Siemens Verio (Siemens Healthcare, Erlangen, Germany). Images included diffusion-weighted imaging or diffusion-tensor imaging, while isotropic diffusion-weighted images and apparent diffusion coefficient (ADC) maps were calculated automatically. To differentiate methaemoglobin from ischemic changes, either T2weighted (w) gradient echo sequences or T1w sequences without contrast agent were obtained.

### Image analysis

**Figure 4:**
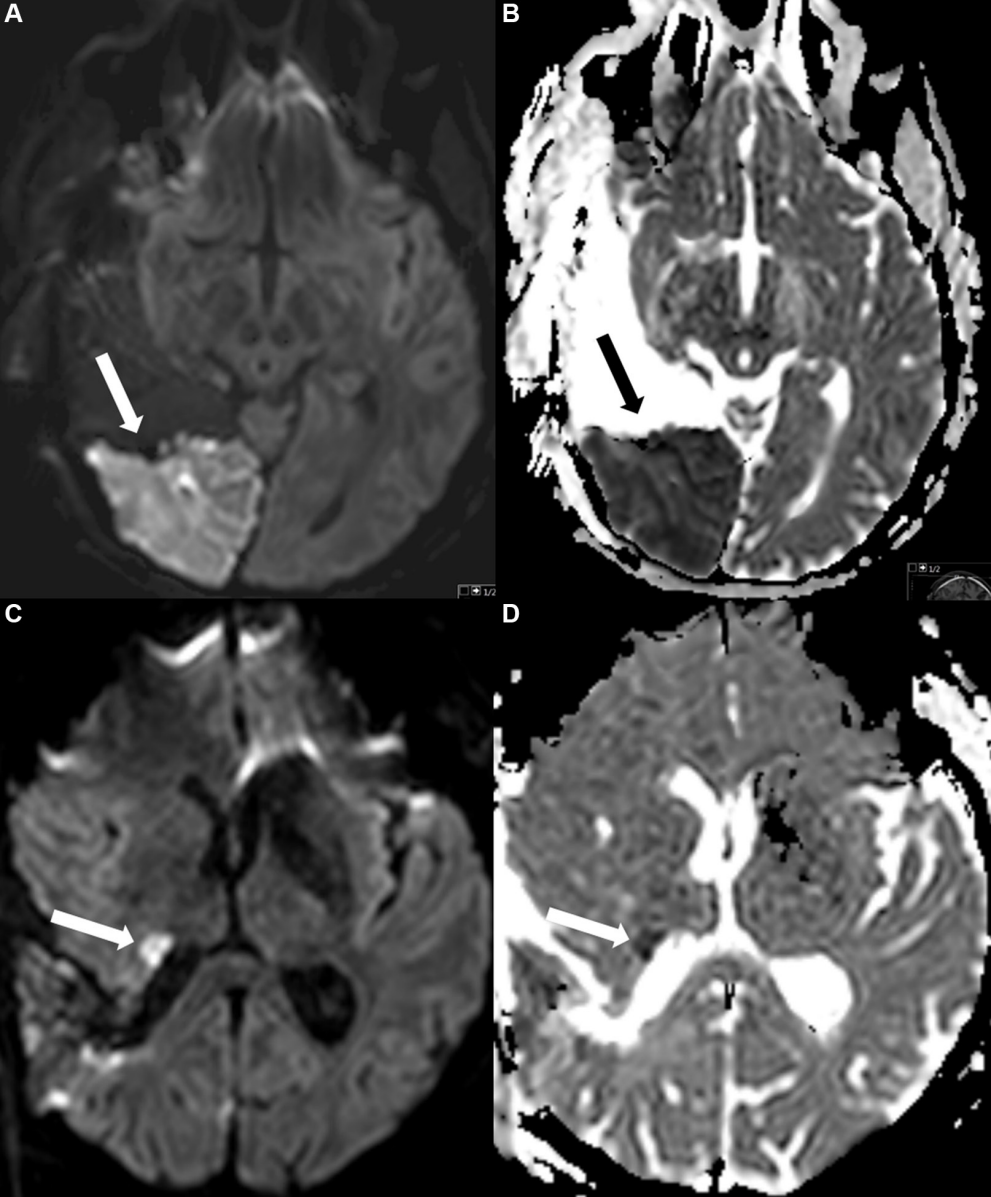
Examples of postoperative ischemic changes The first row (Figure A and B) shows a large area with restricted diffusion (**A**) and corresponding hypointensity in the ADC map (**B**), the second row a smaller area with restricted diffusion (DWI) (**C**) and ADC (**D**) map.

Image analysis was performed by a neurosurgeon (9 years of experience) and a neuroradiologist (6 years of experience) in consensus, blinded to neurological outcome and overall survival. Areas of ischemic lesions were defined by a focal hyperintensity on diffusion-weighted images and a corresponding hypointensity on ADC maps, excluding methaemoglobin and postoperative changes as it was described before [10, 38] (Figure 4).

The volume of the contrast-enhancing tumor part in the pre- and early postoperative MRI and the volume of postoperative infarction were manually segmented using IPlannet (iPlan 3.0 cranial planning software, Brainlab AG, Feldkirchen, Germany). Volumetric measures were based on the Cavalieri principle with unbiased volume estimates [37], as described before [36]. To differentiate methaemoglobin from contrast enhancement in early postoperative MRI, T1w images before and after contrast injection were compared [40]. In two patients measurement of the postoperative tumor volume was not possible due to blurred pictures in 1 case and due to missing contrast agent in the other case.

### Statistical analysis

Statistical analysis, including descriptive data analysis, was performed using IBM SPSS Statistics Version 23.0 (SPSS Inc., IBM Corp., Armonk, NY, USA). Non-normally distributed data are shown as median and interquartile range (IR), normally distributed variables as mean and standard deviation. Correlations were performed using Spearman-rho, differences between groups were analyzed using the Mann Whitney U test for comparison of two groups. Overall survival and PFS distributions were compared using the Kaplan-Meier estimates (Log-rank) and a Cox proportional hazard regression model for multivariate survival analysis. Dichotomization of infarct volume was performed for univariate survival analysis. Iterative data analysis was performed for dichotomization. Differences with a type one error probability of less than 0.05 were considered statistically significant.

## CONCLUSIONS

Postoperative ischemic changes occur frequently after glioblastoma resection. Infarct volume is besides other known factors (age, extent of resection, postoperative KPS) an important prognostic factor for overall survival. These results suggest that hypoxia might be a mediator for invasive tumor growth that is associated with impaired overall survival.

## SUPPLEMENTARY MATERIALS FIGURE



## Abbreviations

DWI: diffusion weighted imaging
FLAIR: fluid attenuated inversion recovery
MPRage: magnetization prepared rapid gradient echo
GB: glioblastoma
ADC: apparent diffusion coefficient
IDH: isocitratdehydrogenase
MGMT: O6-methylguanin-DNA-methyltransferase
PFS: progression free survival
