# Supplementary material for: Infarct volume after glioblastoma surgery as an independent prognostic factor
Source: Oncotarget. 2016 Aug 22;7(38):61945–54. doi: 10.18632/oncotarget.11482 (PMC5308702; doi:10.18632/oncotarget.11482)
Supplement: Supplementary file 1 [file oncotarget-07-61945-s001.pdf]

Infarct volume after glioblastoma surgery as an independent prognostic factor

Supplementary Materials

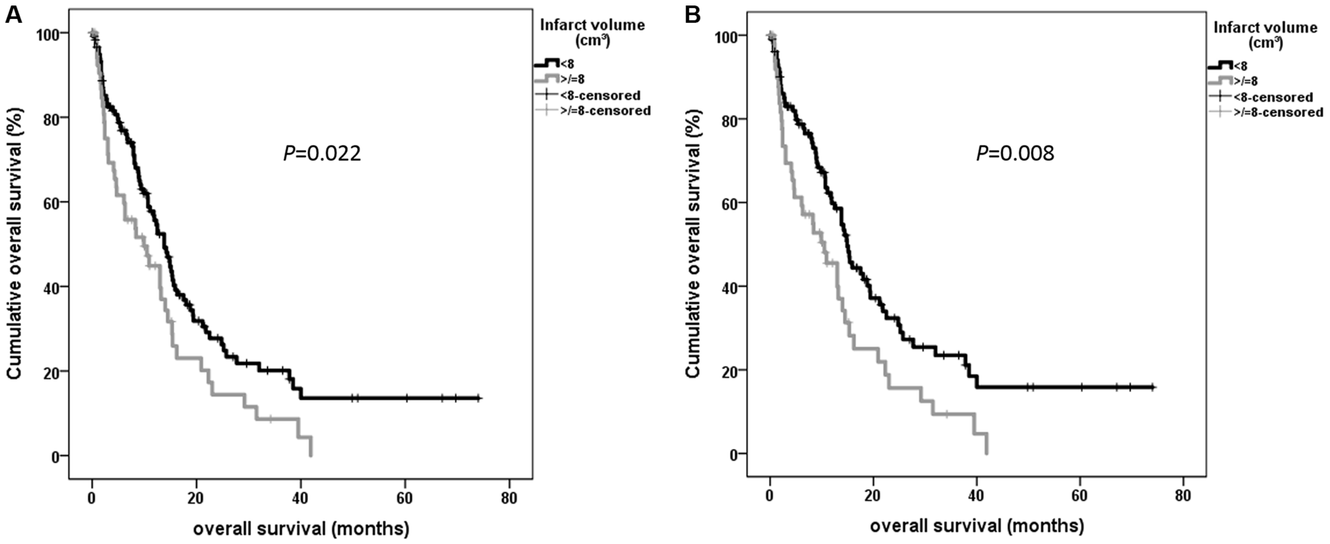

Supplementary Figure S1: Univariate survival analysis using Kaplan-Meier for dichotomized infarct volume for the subgroup of patients without rim-like infarctions (A) and for patients without rim-like infarctions and total and near-total tumor resection (> 90%) (B).
